# Working toward reducing bias in peer review

**DOI:** 10.1016/j.jlr.2021.100124

**Published:** 2021-09-28

**Authors:** Kerry-Anne Rye, Nicholas O. Davidson, Alma L. Burlingame, F. Peter Guengerich

If you are reading this editorial, it is highly likely that you have also read several peer-reviewed publications in the past few weeks. Reading the literature is part of the daily routine for most research scientists. What is rarely uppermost in our thoughts at such times is how these papers transitioned from being a good idea, or a logical extension of previous work, into the final, published product. However, one thing that most scientists do agree on is that much of what we read has been improved enormously by thoughtful and critical peer review. Peer reviewers make an invaluable contribution to manuscripts that are under consideration for publication by journals. This is especially true in the case of submissions that clearly have merit but are incomplete or poorly focused. A high-quality peer reviewer often helps turn these manuscripts into compelling publications that attract the attention of readers. Advances in science also require rigorous validation, and peer reviewers play a vital role in this process.

Excellence in peer review is something that does not come naturally to most of us. It takes a lot of experience to be able to provide authors with succinct and balanced feedback that, if implemented, will significantly improve the quality of a manuscript. Good reviewers unerringly identify gaps and inconsistencies in manuscripts and offer constructive solutions for their resolution. As the reputations of these highly valued reviewers grow, they often find themselves receiving many more requests than they can reasonably be expected to handle, forcing editors to seek out alternate but equally competent reviewers. Building communities of dedicated, expert peer reviewers at all career stages is the key to solving this dilemma and to ensuring a pipeline of quality editorial board members.

We, as editors of the ASBMB journals, are acutely aware of our dependence on the volunteer peer review community for ensuring that we publish quality science. Most manuscripts that are accepted for publication have had input from at least two peer reviewers. An insight into the scale of this effort can be obtained by considering that the three ASBMB journals—*Journal of Biological Chemistry*, *Journal of Lipid Research*, and *Molecular and Cellular Proteomics*— collectively published a total of 1775 peer-reviewed, original research articles in 2020. Assuming that at least two independent reviewers evaluated each of these articles, it follows that a minimum of 3550 volunteer peer reviewers provided input into these publications. As these numbers do not account for submissions that were peer reviewed and not accepted for publication or were re-reviews of revised manuscripts, it is clear we owe an enormous debt of gratitude to our peer reviewer community.

These rather sobering statistics highlight the importance of Peer Review Week, which gives us the opportunity to say a huge thank you to all reviewers who have contributed their time and expertise to evaluating submissions for our journals. Their input ensures that the quality of the science we publish is exceptional and enables us to provide the scientific community with access to reproducible, groundbreaking studies.

In addition to recruiting the best reviewers for the submissions we receive, we have made a concerted effort over the last 2 to 3 years to improve the inclusivity of our peer reviewer community. Strategies we have adopted include inviting outstanding junior researchers to join our editorial boards as early career reviewers. These early career reviewers are mentored by experienced associate editors, giving them the opportunity to develop their reviewing skills. We regard this initiative as a critically important investment in the future of all ASBMB journals, ensuring that we will continue to publish quality and transformative science for the foreseeable future.

We are also evaluating additional strategies for improving the equity, diversity, and inclusivity of our peer review communities, a topic that is directly aligned with the focus of this year's Peer Review Week. Bias in peer review has attracted increased attention in recent years. It is pervasive, goes largely undetected, and has only recently been openly acknowledged as a significant issue (1). Reducing bias in peer review is critically important to ensure the integrity of our editorial process and embrace diversity, equity, and inclusion in our scientific communities. As a practical matter, we will encourage associate editors to take gender, racial, and geographical diversity into consideration when selecting reviewers for submitted manuscripts. The importance of bias awareness is highlighted in recent publications that have established that both male and female editors have a clear preference for peer reviewers of the same gender and from the same country (1, 2).

This is also the case for ASBMB journals. How do we, as editors, address this? In the first instance, we will be regularly reminding the editorial teams about the potential for bias. As a first step the JLR editors-in-chief have decided to provide all associate editors with regular, individualized, geographical and gender distribution summaries of the peer reviewers they are using. We will also be focusing on increasing inclusion of underrepresented minority groups in the peer review community. The JLR has already gone some way toward addressing this with the appointment of members of underrepresented minorities as junior associate editors.

While we believe that these actions will help reduce peer reviewer bias in ASBMB journals, we realize that they fall short of resolving it. Going forward, we will be regularly reviewing demographic and other data that relate to our peer review communities and continue to work toward reducing all aspects of bias. It is especially important that these changes are implemented in ways that do not compromise the quality of the science we publish. Although there is no doubt that some of these changes will create challenges, their implementation has the potential to bring major benefits to all ASBMB journals and promote the development of a large, highly diverse, and insightful peer review ecosystem that should serve as a role model for peer review across the broader scientific community.
